# The Role of Dehumanization in Our Response to People With Substance Use Disorders

**DOI:** 10.3389/fpsyt.2020.00372

**Published:** 2020-05-15

**Authors:** Teneille R. Brown

**Affiliations:** ^1^Center for Law and the Biomedical Sciences, University of Utah, Salt Lake City, UT, United States; ^2^S.J. Quinney College of Law, Center for Law and the Biosciences, Program in Medical Ethics and Humanities, The University of Utah, Salt Lake City, UT, United States

**Keywords:** addiction, harm reduction, dehumanization, stigma, chem sex

## Stigma and Dehumanization Surrounding Addiction

The way we respond to substance use disorders (SUD) says more about our society than it does about those using illicit drugs. In the United States, in particular, the very distinction between illicit and legal use depends on politics and power more than a drug’s specific risk profile. While inconsistently penalized, drug use has largely been framed as a criminal justice issue. This framing has fueled, and been fueled by, the intense stigma surrounding addiction. The process of stigmatization relies on sticky overgeneralizations, which in turn are used to justify social exclusion, prejudice, and discrimination. Addiction is one of the most stigmatized social disorders, with “addicts” being described as “filthy junkies” who do not deserve social support or empathy (1). A recent study found that nearly 73% of those surveyed would be unwilling to spend even *one* evening socializing with an “addict.” (2) This social distancing contributes to the low self-esteem and depression people with SUD experience. It can also foster punitive policies such as insurance coverage disparities and housing discrimination (3).

Stigma also relates to dehumanization, which is when we deny out-groups human characteristics that we extend to ourselves—namely, the capacity to feel and the capacity to make decisions. Throughout history when groups are dehumanized, they are seen not as individuals but as members of a mindless cluster to whom we can direct our moral outrage and punishment (4). On scales of dehumanization, drug addicts often rate as “the lowest of the low,” (5) triggering reactions of disgust due to their perceived unpredictability and lack of agency. This can even be evidenced at the neurological level, as viewing images of people with SUD does not activate regions of the brain normally recruited when viewing humans (6).

## Differing Levels of Moral Outrage Mediate Our Response

Given that stigma and dehumanization take root when we fail to take the perspective of the affected person, it is not surprising that this lack of empathy is exaggerated when the particular type of “addict” is dissimilar to us. This may be why the level of collective disgust and moral outrage shown to people with SUD has been inconsistent over time. Our moral outrage, and not our public health priorities, determine our social response. We need look no further than the recent history regulating drug use in the United States for confirmation of this.

### Crack Cocaine and the “War on Drugs”

In the 1970s, the racialized “war on drugs” began with poor black communities placed in the government’s crosshairs. Congress passed harsh mandatory minimum sentences, that decimated communities of color. These laws were supported by dehumanizing media portrayals of black “crack whores” and black “crack babies.” (7) Even when the drug was pharmacologically identical, our criminal and public health response focused not on the harms but on the population affected. While powder cocaine, “associated with a wealthier, whiter class of drug users,” required possession of 500 grams to trigger a 5-year federal prison term, one only needed to possess 5 g of crack cocaine, “regarded as a drug of the black urban ghetto,” to trigger the same sentence (8). Drugs rarely stay in the hands of a discrete subpopulation and tend to spread to many other communities. However, the initial framing of the using population can steer our legal and public health response.

### The Modern Opioid Crisis

Today, the opioid crisis has exposed the truth that addiction does not discriminate between the powerful and the poor. Many suburban, white, teenagers began their opioid addiction in a doctor’s office, filling pain prescriptions before turning to stronger drugs (9). Now, most opioid deaths in the United States are due to fentanyl overdoses (10). While still infused with criminalization, many agencies and legislators have responded to the crisis with a more therapeutic and prevention-based approach, that recognizes the humanity of those affected (11). Congress even passed a rare, bipartisan appropriation bill that provided funding for research into addiction stigma, and increased housing options for people in recovery (12). Politicians have thankfully also taken stock of the structural roots of the crisis: physician conflicts of interest, underregulation of greedy pharmaceutical companies, poverty, and a lack of access to quality mental health care. In part because of the devastating economic impacts, local governments are even attempting to hold all of the participants in the supply chain responsible through tort litigation, rather than focusing blame on the end user (13). These delayed but significant efforts laudably treat addiction as a disease with devastating public health impacts. However, it is not coincidental that this addiction crisis struck the white suburbs before the urban poor, which framed, and likely facilitated, the more empathic response (14).

### The Use of Cognitive Enhancers

Cognitive enhancement is the nontherapeutic use of drugs to boost memory, attention, or alertness. While the fatality risk from stimulants like methylphenidate and amphetamine (sold as Ritalin and Adderall) is lower than for opioids or cocaine, there is still considerable, underexplored risk of their off-label use (15). People can become dependent or addicted to these substances, and in high doses they can cause toxicity and cardiac arrest. The perceived market for these study drugs is largely young, privileged, college students, who are looking for a competitive edge (16). And while many see this use as unfair and troubling (and indeed illegal if the result of diversion), it has not triggered the same level of moral outrage. There is a growing market for such drugs on the dark web. However, cognitive enhancement is still chiefly identified with college campuses. This framing has enabled a more libertarian approach. The regulatory response has been mainly left to university administrations, a minority of which have declared the use of cognitive enhancers a violation of their honor codes. This can be understood through the lens of dehumanization—privileged, white, college students are considered to have maximum levels of agency and emotionality. They are thus granted the status of full humans, similar to those who were tricked into being addicted to prescription pain medication, but unlike those lesser humans who willingly became addicted to crack cocaine. The consequence of this are startling. Being held to have violated a university honor code and being labeled a “cheater” is a far cry from a mandatory prison sentence of up to 5 years and a lifetime label as “convict.”

### “Chem Sex” and a Chance to Do Better

The use of drugs before, during, or after sex is not new. However, sexualized drug use, or “chem sex” has recently been increasing among men who have sex with men (MSM), and is linked with high-risk sexual behaviors such as condomless anal sex and the transmission of sexually transmitted infections (17). The drugs consumed vary according to geography and access, but common combinations include mephedrone, GHB, methamphetamine, and drugs for erectile dysfunction (18). These drugs may be taken to reduce sexual inhibitions, stimulate arousal, intensify orgasm, or keep individuals awake. The risks associated with this practice depend on the frequency and patterns of use, and drug-drug interactions (19). For example, the effects of GHB are exaggerated in people on certain HIV medications, and chem sex can reduce compliance with these protocols.

So far, our societal response to chem sex has been a bit muted. Insufficient epidemiological data has been gathered to describe its prevalence and features. There are vulnerable intersectionalities at play, as drug users and gay men are both dehumanized. Gay men are routinely dehumanized with metaphors of deviance and animalistic sexual practices which deny their status as fully thinking and feeling individuals. Indeed, this might contribute to their chem sex drug use—as they may be isolated, anxious, and unprepared to negotiate their sexual lives in a world that does not allow them to develop this openly. Chem sex, like other drug use, might be a form of self-medication. Referring to “chem sex” as a “second plague” which is “unnatural” and filled with “animalistic orgies” perpetuate this hypersexualized and monolithic narrative.

We have a chance to do better when it comes to our response to chem sex. While the public health concerns are already mounting, it is not too late to frame our response in a way that maximizes the humanity of those affected. We can start with using less sexualized terminology that focuses on the environment in which people use. Just as we are beginning to do with those with opioid use disorder, we must invest in harm reduction measures and evidence-based prevention and treatment strategies (20). Encouraging group use of drugs can be a method of harm reduction rather than a means for stigmatization, as individuals can monitor each other for signs of cardiac arrest or shallow breathing. Legislators should consider enacting “Good Samaritan” statutes, which immunize fellow users from prosecution if they call emergency responders when someone overdoses. Many states have passed such laws in the wake of the opioid crisis, but they should be applied to the same extent when MSM engage in chem sex. It is unlikely that chem sex will be dismissed as an autonomous exercise of agency, as many unfortunately do with the enhancement uses of stimulants. Without denigrating those who engage in chem sex as subhuman, we should recognize that there are serious potential harms that flow from this choice. These harms must be shared in a nonjudgmental way with affected communities. We must better understand the causal relationships between risky sex and drug use, and explore the reasons why people use, and how they are using, to understand the best paths toward encouraging healthy behaviors (21). None of these strategies will be effectively pursued if we ignore the humanity of those affected, or allow the sexual nature of this practice to stifle good epidemiological research. Substances have different risk factors for abuse, which cannot be ignored. But each underlying population is completely human—with complete capacity to think and feel. It does violence to our prevention and treatment efforts when we allow our social response to be guided by sympathy for those “like us” and antipathy for those who are different.

## Author Contributions

TB is the sole author and contributed 100% to the research and writing of this Opinion.

## Conflict of Interest

The author declares that the research was conducted in the absence of any commercial or financial relationships that could be construed as a potential conflict of interest.
